# Pseudocholinesterase Deficiency – Is Succinylcholine Still Needed to Facilitate Endotracheal Intubation?

**DOI:** 10.7759/cureus.10721

**Published:** 2020-09-29

**Authors:** Lakshmi N Kurnutala, Nickhil Rugnath

**Affiliations:** 1 Anesthesiology and Perioperative Medicine, University of Mississippi Medical Center, Jackson, USA; 2 Anesthesiology, University of Mississippi Medical Center, Jackson, USA

**Keywords:** succinylcholine, pseudocholinesterase deficiency, rocuronium, sugammadax

## Abstract

Pseudocholinesterase (butyrylcholinesterase) deficiency is an inherited or acquired condition in which the serum pseudocholinesterase levels are absent or lower than normal. The enzyme is produced by the liver; decreased levels of the enzyme in an individual cause increased sensitivity to anesthetic agents, like succinylcholine and mivacurium. Pseudocholinesterase deficiency is caused by butyrylcholinesterase (BCHE) gene mutation, a gene that provides instructions for making the pseudocholinesterase enzyme. Succinylcholine is a depolarizing muscle relaxant that provides a quicker onset and a brief duration of muscle relaxation during general anesthesia. In this article, we would like to discuss a case report of prolonged intubation and ventilation in a patient with pseudocholinesterase deficiency and the necessity of succinylcholine during intubation in comparison to possible alternatives (rocuronium).

## Introduction

Pseudocholinesterase (butyrylcholinesterase) is an enzyme produced in the liver and is responsible for the metabolism of several common anesthetic drugs, including mivacurium and succinylcholine. In individuals with normal serum enzyme levels, it metabolizes succinylcholine and the duration of action of the medication is less than 10 minutes [1-2]. In an individual with a pseudocholinesterase deficiency, there is a reduced ability to efficiently metabolize these anesthetic medications which results in the prolonged effect of neuromuscular paralysis [3-4]. This prolonged neuromuscular blockade requires intensive care unit (ICU) or post-anesthesia care unit (PACU) stay for mechanical ventilation until normal breathing resumes. A longer stay in the hospital increases the economic burden on the patient and the hospital [3].

## Case presentation

An 18-year-old Caucasian female (body mass index (BMI) - 28 Kg/m^2^) was scheduled for a revision of a bilateral breast reduction under general anesthesia. The patient had no known allergies and was not currently taking any medications. The patient had no prior medical history (American Society of Anesthesiologists (ASA) physical status 1). Her past surgical history included breast reduction surgery one year prior with no anesthetic complications. In the operating room, the patient was connected to standard ASA monitors. General anesthesia was provided with midazolam (2 mg), propofol (150 mg), fentanyl (100 mcg), and succinylcholine (100 mg), and it was maintained with sevoflurane, oxygen, and air. The surgical procedure lasted for one hour with no additional opioids intraoperatively. At the conclusion of the surgery, the patient exhibited no twitches (0/4) and no respiratory effort. The patient was treated with one dose of intravenous naloxone (80 mcg) with the possibility of opioid sensitivity; there was no change in the patient’s respiratory effort. The patient became increasingly hypertensive and tachycardic as the sevoflurane wore off. The patient's preoperative labs were within normal limits. Because of this, it was suspected that the patient had a pseudocholinesterase deficiency. The patient was re-sedated with midazolam and propofol, transferred to the PACU for ventilator support. The patient was extubated four hours later after meeting the extubation criteria. A blood sample was drawn for the pseudocholinesterase levels from the patient which showed a serum level of 0.2 U/ml (normal - 1,800 - 6,600 U/L). The patient was observed for 24 hours, informed about the pseudocholinesterase deficiency, and discharged home the following day with no further complications.

## Discussion

Atypical pseudocholinesterase deficiency incidence and tests

Pseudocholinesterase deficiency is an uncommon condition that is present in about 1 in 3,200 to 5,000 people [1-2]. Although it is an autosomal recessive genetic condition, this condition is more prevalent in certain populations, including the Persian Jewish community and Alaskan natives. A mutation in the butyrylcholinesterase (BCHE) gene causes the formation of an abnormal pseudocholinesterase enzyme, resulting in pseudocholinesterase deficiency [5]. This condition is inherited as an autosomal recessive trait located on chromosome 3 (3q26.1-26.20), skipping generations as affected individuals are children of unaffected parents [6]. Heterozygotes for this condition do produce a mid-level response compared to the homozygous dominant and homozygous recessive individuals. Although many individuals may be suspected of pseudocholinesterase deficiency or with a family history of prolonged action to succinylcholine, testing can be done prior to surgery to determine if an individual has the pseudocholinesterase deficiency condition [7]. These preoperative tests/indicators include genetic history, prior medical history, pseudocholinesterase enzyme levels, dibucaine number, and fluoride number. Acquired causes of pseudocholinesterase deficiency include chronic kidney disease, liver failure, malnutrition, major burns, malignancy, medications, pregnancy, and after cardiopulmonary bypass [6].

Role of succinylcholine in emergent intubation

Succinylcholine first introduced in 1949. It is a depolarizing muscle relaxant used for rapid induction and intubation during general anesthesia [1]. Although the rapid onset of succinylcholine allows for rapid onset of intubating conditions unprecedented from other alternatives, succinylcholine has an ultra-short duration of effect compared to the alternatives available. Succinylcholine works as an acetylcholine agonist, mimicking the action of acetylcholine by depolarization of the post-junctional membrane [8]. Succinylcholine’s mechanism works by the persistent depolarization and subsequent blockage of the nicotinic postsynaptic acetylcholine receptors in the neuromuscular junctions. The neuromuscular blocking agents (NMBA) provides a consistent block, allowing for rapid intubating conditions, during the elective or emergency airway management. Because of multiple complications related to succinylcholine, more anesthesiologists reduced its use in emergency and elective intubation and with the availability of newer, nondepolarizing muscle relaxants with lesser side effects [9-10]. The Food and Drug Administration (FDA) issued a black box warning in 1993 for succinylcholine after a series of cardiac arrests occurred related to hyperkalemia in children with undiagnosed muscular dystrophy [11-12]. Cardiac arrest related to hyperkalemia also occurs in adults with a history of stroke, burns, prolonged immobilization, and spinal cord injury. Other side effects related to succinylcholine are malignant hyperthermia, masseter spasm, myalgia, anaphylaxis, increase intracranial pressure (ICP), and intraocular pressure [1, 4].

Rocuronium and sugammadex in emergency intubation

Rocuronium is a non-depolarizing muscle relaxant that facilitates intubating conditions during general anesthesia and provides adequate muscle relaxation to facilitate surgical procedures. Rocuronium works by blocking the alpha subunits nicotinic acetylcholine receptor of the neuromuscular synapse, blocking any possibility of depolarization or the conduction of action potential [1, 4, 13]. Rocuronium has a longer onset time (90 sec with 1.2 mg/kg body weight) and a long duration of action (30 - 40 minutes) than succinylcholine [14]. In a study done by Patanwala et al. in the emergency department, succinylcholine and rocuronium provided equal intubating conditions and first attempt success [15]. The effects of rocuronium are reversed with the administration of sugammadex, a neuromuscular reversal drug. Sugammadex is a 72-carbon compound that is joined through 1-4 glycosyl bonds produced from starch bonds [16]. Sugammadex mechanism of reversing the effects of rocuronium is to chelate the free molecule to form a more stable complex [17]. The forming of this tight complex causes a change in the free rocuronium gradient, causing the free rocuronium to move from the tissue into the plasma of the individual [18]. The movement of rocuronium into the plasma allows the reduction of the muscle relaxation effect, restoring normal contraction in the individual [11]. The dose of sugammadex (2 - 16 mg/kg body weight) is based on the number of twitches on neuromuscular monitoring (train of four - TOF). After giving rocuronium as a part of the general anesthesia with TOF monitoring, in the presence of two twitches (T2), the dosage of sugammadex (2 mg/kg body weight) reverses the neuromuscular blockade (NMB). If the patient had no twitches on TOF monitoring, but in presence of one to two post-tetanic contractions (PTCs), sugammadex 4 mg/kg provides reversal of NMB. In emergencies, like 'can’t ventilate' or 'can’t intubate' situations, to reverse the muscle relaxant effect of rocuronium, 16 mg/kg dosage is used [12]. The disadvantage of rocuronium is a higher incidence of anaphylaxis (1:22,000), but the incidence is not higher than succinylcholine (1:2000) [19]. Rocuronium duration of action was prolonged in patients with renal disease [14].

## Conclusions

The necessity of succinylcholine has come into question because of the possibility of a patient with pseudocholinesterase deficiency and the side effects it produced. With the use of rocuronium, the factor of an individual having pseudocholinesterase deficiency becomes irrelevant because of it being a non-depolarizing muscle relaxant. Succinylcholine during general anesthesia provides the rapid onset of intubating conditions and a short duration of action. However, with the current availability of sugammadex as a reversal agent, rocuronium has become a viable alternative to produce similar conditions with fewer side effects and avoid prolonged ventilation in patients with pseudocholinesterase deficiency. 
